# Clinical and physiological evaluation of free-ranging snow leopards immobilized with ketamine-xylazine in emergency situations

**DOI:** 10.3389/fvets.2025.1492640

**Published:** 2025-02-25

**Authors:** Animesh Talukdar, Anchal Bhasin, Dimpi Patel, Pankaj Raina, Prasad Tonde, Prateek Savita

**Affiliations:** ^1^Department of Wildlife Protection, Leh, UT-Ladakh, India; ^2^Amity Institute of Environmental Science, Amity University, Noida, Uttar Pradesh, India; ^3^Wildlife Institute of India, Dehradun, Uttarakhand, India

**Keywords:** immobilization, *Panthera uncia*, snow leopard, physiological, clinical, reversal

## Abstract

The current study presents data on the immobilization and physiological responses of 26 distressed free-ranging snow leopards (*Panthera uncia*) in the trans-Himalayan regions of Ladakh, India, spanning three years from October 2020 to December 2023. Ketamine and xylazine were utilized in a drug mixture for rescue, rehabilitation, health assessment, and other capture purposes, with average doses of 6.535 ± 0.93 mgkg^−1^ and 1.937 ± 0.41 mgkg^−1^ of body weight, respectively. The average induction occurred at 3.85 ± 1.8 min. Respiratory rate, rectal temperature, and heart rate were monitored periodically post-induction, all remaining within clinically acceptable ranges. Following an average recumbency period of 70.69 ± 16.56 min, immobilizations were reversed using intramuscular injections of Yohimbine at 0.147 ± 0.03 mgkg^−1^ of body weight, leading to complete recovery within an average time of 24.92 ± 7.08 min. Our findings suggest that the ketamine and xylazine mixture represents a safe and effective method for immobilizing snow leopards, particularly in emergency scenarios.

## Introduction

The snow leopard (*Panthera uncia*), known for its elusive nature and high-altitude habitat, faces significant threats throughout its range (1). According to the International Union for Conservation of Nature (IUCN) Red List, the snow leopard is currently listed as vulnerable, with population trends showing a decline (2). Distributed across Asian high mountain habitats in 12 countries, the snow leopard is a flagship species for conservation efforts in the Indian Himalayan region (3).

Although non-invasive ecological research methods are increasingly used to inform snow leopard conservation, there are still good reasons to develop protocol that conducts research and conservation that requires safely capturing, immobilizing, and releasing snow leopards (4), including telemetry-based studies (5–8, 27–29) morphometric studies (9, 10), managing conflict situations and health assessments (11, 12). Such investigations are necessary to know their activity pattern, health conditions, emergency health conditions, and other physiological details (4, 12).

Emergencies for wildlife rescue can range from natural disasters, accidents, mass relocation, etc. to closed human-wildlife interaction. Carnivores, when stressed in any of such conditions tend to be aggressive hence chemical immobilization is implemented for the safety of the animal and the personnel involved in the rescue operation. Chemical immobilization is a necessary component of conservation and management activities that involve capturing and handling snow leopards, when properly used it is safe and causes far less stress to captured animals (13). Ketamine-Xylazine immobilizations have been used historically for the immobilization of many species; both domestic and wild, including other free-ranging threatened carnivores (14). Similarly, Yohimbine has also been used historically as an antagonist against Xylazine for reversal in a variety of animal species (13). Various drug mixtures have been used to immobilize both captive and free-ranging snow leopards, including tiletamine-zolazepam, medetomidine-ketamine, ketamine-xylazine, and medetomidine-tiletamine-zolazepam, but published information on the drug doses, clinical and physiological response of free-ranging snow leopards remains limited (15–18). Given the diversity of legal restrictions across the 12 countries that snow leopards inhabit, India has higher legal restrictions; thus, legal field-immobilizing drugs are limited to ketamine hydrochloride and xylazine hydrochloride (19, 20). There is a need to evaluate and share information available to help wildlife health professionals develop safe and flexible immobilization protocols. Therefore, our objective was to assess ketamine-xylazine drug combination immobilization’s physiological and clinical effects in free-ranging snow leopards in distress based on a standard set of clinical parameters to aid future research and conservation efforts of this iconic feline.

## Materials and methods

### Ethics approval statement

Not applicable. The study was carried out by compiling and analyzing data collected during routine rescue operations permitted by the Department of Wildlife Protection, Leh, Ladakh India. All the authors agree to participate in the publication. The work is presented for publication in the Frontiers in Veterinary Science – Anesthesiology and Animal Pain Management journal.

### Study area

**Figure 1:**
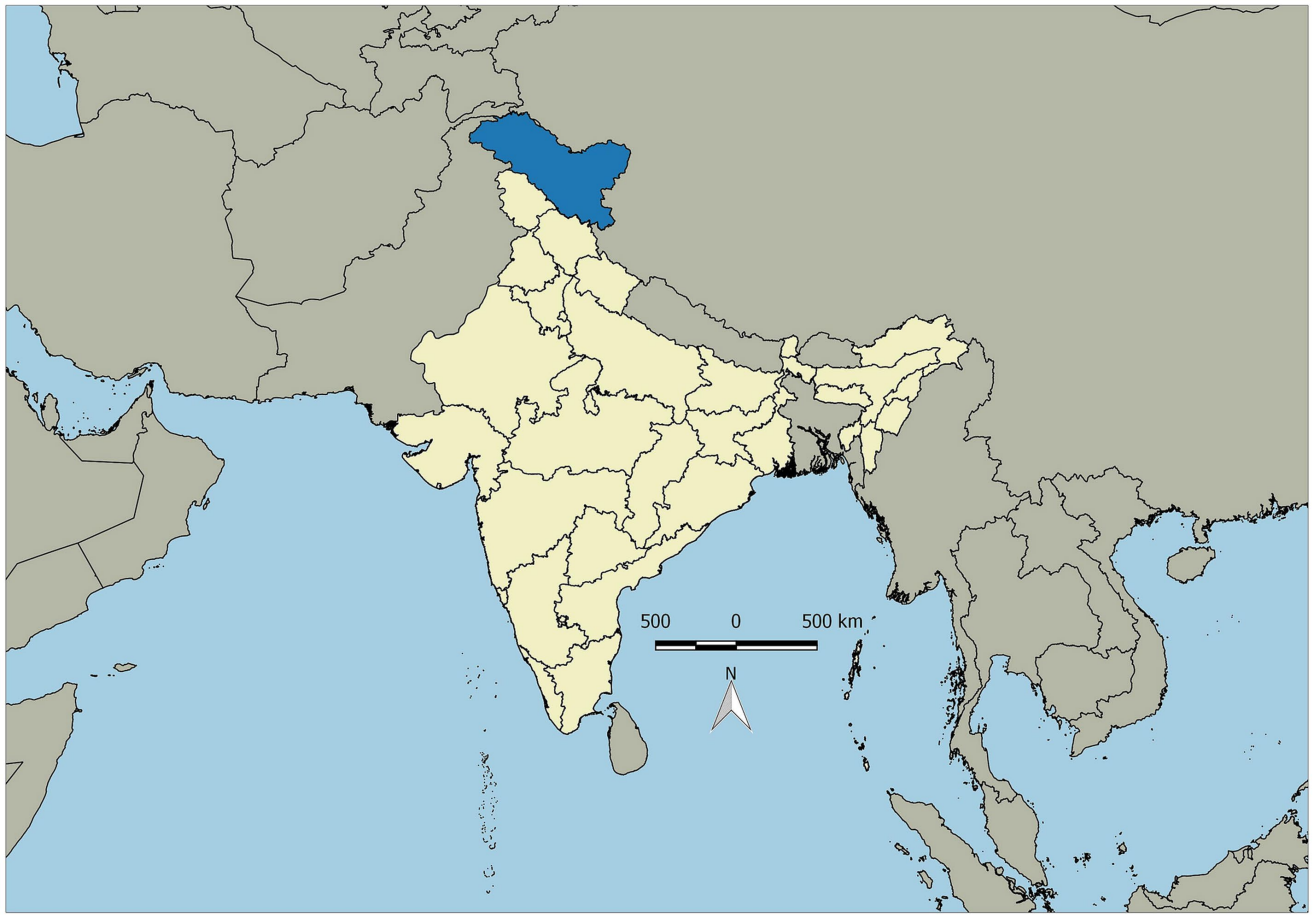
Map of the study area, Union Territory of Ladakh, India.

The study was conducted in the protected areas of Leh, Ladakh, which include Hemis National Park, Karakoram Wildlife Sanctuary, and Changthang Wildlife Sanctuary, as well as their adjacent areas (Figure 1). These areas fall under the western Trans-Himalayan region of India, with a total area of over 78,000 km^2^ and an altitudinal range of 2,700–7,560 m above sea level. The study area is located between longitudes of 32°15′ to 34°38′ N and latitudes of 75°36′ to 78°22′ E.

### Study animal capture and immobilization

Between October 2020 to December 2023, 26 free-roaming snow leopards (11 males, 15 females), mean ± SD values for body mass was 29.69 ± 4.35 kg (21–40 kg) were immobilized to facilitate rescue and rehabilitation efforts from human-wildlife conflict situations linked to livestock depredation and displacement. In most cases of livestock depredation, the snow leopards were trapped by livestock owners inside animal shelters, who then informed the forest department for rescue, which promptly dispatched a rescue team. The time required to reach the rescue site depended on its location, but all rescues were carried out within eight hours of receiving the alert.

Since animals were trapped near human habitation as mentioned earlier, it was necessary to immobilize them chemically to reduce the risk of injury to animals and humans.

All the snow leopards were chemically immobilized using ketamine (KETAMINA, 100 mg/mL, Biowet Pulawy, Poland or VETALAR, 100 mg/mL, Parke Davis & Co., P.O. Box qq8 GPO, Detroit, Michigan 48,232, USA) in combination with xylazine (XYLAMED, 100 mg/mL, Bimeda, Cambridge, Ontario) at dose rates of 6 mg/kg and 1.5 mg/kg, respectively. In field or emergencies where the exact weight of the animal could not be determined before immobilization, the dosages loaded into the dart were based on estimated weight, approximated from the animal’s apparent size. Factors such as the animal’s health condition (e.g., if injured or stressed), state of excitement, and size were also considered. In cases where the animal was hurt or experiencing high stress, higher doses were selected to ensure rapid immobilization and for animals in a highly excited state. Smaller animals were often given relatively higher doses per estimated weight to account for quicker metabolism and stress responses. Once immobilized, the actual weight of the animal was recorded during processing, allowing for retrospective assessment of the dosage per kg of actual body weight. Initial dosages of 6 mg/kg and 1.5 mg/kg were estimated, with adjustments and additional doses administered if full immobilization was not achieved after the first attempt, based on the animal’s response. All individuals were healthy, and chemical capture was chosen accordingly. Captures were carried out in temperatures ranging from 28°C to −20°C. Darting was conducted on foot from distances ranging from 5 to 20 meters. The drug combination was administered remotely via a 3 mL dart into the right or left quadriceps muscle of each animal.

The immobilizing drug mixture was remotely administered using air-pressurized syringe projectors [Daninject CO_2_ Injection Rifle Model IM (DAN-INJECT ApS, Kolding, Denmark)]. Due to the availability of higher concentrations of drugs as 100 mgmL^−1^ for both ketamine and xylazine, 3 mL nylon darts were adequate with needle lengths of 30 mm and diameter of 1.5 mm for drug administration (N1530 needle, 17G x 1.25″ (1.5 mm x 30 mm).

### Animal handling and monitoring

The free-ranging condition and rescue situation at times did not provide opportunities for documentation of all the events of drug induction and the recordings were limited to information after locating and capturing the animal. For each snow leopard, once it was determined that anesthesia was complete and it was safe to approach and handle the animals, we blindfolded, weighed using an industrial grade circular hanging spring balance (Capacity 25 kg – 100 kg), assessed health condition, performed treatment if necessary and collected tissue samples, while monitoring physiological parameters throughout. Rectal temperature (RT), heart rate (HR), and respiratory rate (RR) were monitored every five minutes after induction for 30 min whenever possible, as most of the cases were for rescue operations and with human presence nearby after 30 min either reversal was administered for all the cases or kept inside transportation box within 30 min to ensure safety for human as well as for the animals. Based on tooth wear colour and body size, age was estimated for all the animals captured (5, 10).

### Drug reversal

Drug reversal was facilitated by administering yohimbine (YOHIMBE, 10 mg/mL, Equimed USA) at a dose rate of 0.125 mgkg-1 body weight intramuscularly. We subsequently monitored the time post-reversal to eye and head movement followed by ‘able-to-stand’ time.

### Data analysis

All the recorded data for the drug dose, induction, and reversal time along with clinical parameters and physiological variables were evaluated for normal distribution using the Shapiro–Wilk test. The data for the differences within each recorded variable between sex and age class were assessed separately due to the limited sample size. Since the data was not normally distributed, non-parametric tests (Mann Whitney U test) were conducted. All the statistical analyses were performed with a 95% level of significance using SPSS software (IBM Corporation, USA). Mean, standard deviation, confidence intervals (95%) along with sample size are reported for the entire sample set, and data are presented as Mean ± SD unless otherwise stated.

## Results

All 26 captures showed complete immobilization with no adverse effect (such as hypothermia, hypothermia, tachycardia, bradycardia and deep or superficial respiration) was observed from any of the animal captured. Although physiological and clinical parameters (induction time, recumbency period, complete recovery period) were recorded for most snow leopards, for some individuals, parameters such as heart rate, rectal temperature, and respiratory rate could not be collected at 5 min intervals owing to challenging rescue and field situations including extreme climatic conditions (Tables 1–3).

**Table 1:** Drug doses, physiological parameters, and clinical responses of immobilized snow leopards (*Panthera uncia*) with xylazine-ketamine in Ladakh, India.

Parameters	Unit	*N*	Mean ± SD	CV (%)	Central 95% interval	Range
Xylazine HCL	mgmL^−1^	26	1.937 ± 0.41	0.212	1.77–2.10	1.34–2.86
Ketamine HCL	mgmL^−1^	26	6.535 ± 0.93	0.142	6.16–6.91	4.41–8.33
Induction time	Minute	26	3.85 ± 1.8	0.469	3.12–4.57	2–10
RR 05 (on approach)	/minute	26	30.65 ± 4.39	0.143	28.88–32.43	24–40
RR 05–10	/minute	26	29.69 ± 3.22	0.109	28.39–30.99	22–36
RR 10–15	/minute	26	28.64 ± 2.98	0.105	27.26–29.67	24–36
RR 15–20	/minute	26	29.27 ± 2.62	0.089	28.21–30.33	25–35
RR 20–25	/minute	26	30.27 ± 2.69	0.089	29.18–31.36	25–36
RR 25–30	/minute	26	29.77 ± 1.88	0.063	29.01–30.53	28–34
HR 05 (on approach)	/minute	26	73.73 ± 11.95	0.162	68.90–78.56	60–98
HR 05–10	/minute	26	73.00 ± 10.7	0.147	68.68–77.32	58–95
HR 10–15	/minute	26	72.85 ± 10.25	0.141	68.71–76.99	58–90
HR 15–20	/minute	26	73.19 ± 10.91	0.149	68.78–77.60	60–96
HR 20–25	/minute	26	72.27 ± 9.28	0.131	68.42–76.05	60–90
RT (on approach)	°C	26	38.23 ± 0.48	0.011	37.76–38.7	36.66–39.27
RT 05–10	°C	26	38.23 ± 0.48	0.013	35.1–39.33	37.22–39.27
RT 10–15	°C	26	38.19 ± 0.41	0.012	37–39.44	37.5–38.95
RT 15–20	°C	26	38.23 ± 0.37	0.011	37–39.44	37.5–39.17
Recumbancy period	minute	26	70.69 ± 16.56	0.234	64–77.38	48–120
Yohimbine	mgkg^−1^	26	0.147 ± 0.03	0.203	0.134–0.158	0.088–0.20
Complete recovery (After reversal)	minute	26	24.92 ± 7.08	0.284	22.06–27.78	15–40

**Table 2:** Differences (*α* = 0.05) in drug doses, clinical and physiological parameters between adult and young snow leopards (*Panthera uncia*).

Variables	Unit	Adult		Young		*p*-value
		*n*	Mean ± SD	*n*	Mean ± SD	
Drug doses						
Xylazine HCL	mgkg^−1^	16	1.916 ± 0.47	10	1.971 ± 0.31	>0.05
Ketamine HCL	mgkg^−1^	16	6.221 ± 0.65	10	7.04 ± 1.10	<0.05
Yohimbine	mgkg^−1^	16	0.153 ± 0.02	10	0.136 ± 0.03	>0.05
Clinical parameters						
Induction time	Minute	16	4.13 ± 2.16	10	3.40 ± 0.96	>0.05
Recumbancy period	Minute	16	70.94 ± 12.89	10	70.30 ± 22.01	>0.05
Complete recovery (After reversal)	Minute	16	25.44 ± 7.02	10	24.10 ± 7.47	>0.05
Physiological parameters						
RR 05 (on approach)	/minute	16	30.06 ± 4.84	10	31.60 ± 3.59	>0.05
RR 05–10	/minute	16	29.19 ± 3.25	10	30.5 ± 3.17	>0.05
RR 10–15	/minute	16	28.75 ± 3.06	10	28.00 ± 2.94	>0.05
RR 15–20	/minute	16	29.63 ± 2.94	10	28.70 ± 2.00	>0.05
RR 20–25	/minute	16	30.38 ± 2.91	10	30.10 ± 2.42	>0.05
RR 25–30	/minute	16	30.13 ± 2.09	10	29.20 ± 1.39	>0.05
HR 05 (on approach)	/minute	16	72.56 ± 12.74	10	75.60 ± 10.94	>0.05
HR 05–10	/minute	16	71.31 ± 10.44	10	75.70 ± 11.11	>0.05
HR 10–15	/minute	16	71.25 ± 10.09	10	75.40 ± 10.50	>0.05
HR 15–20	/minute	16	72.13 ± 10.85	10	74.90 ± 11.37	>0.05
HR 20–25	/minute	16	71.00 ± 8.56	10	74.20 ± 10.89	>0.05
RT (on approach)	°C	16	38.13 ± 0.57	10	38.26 ± 0.41	>0.05
RT 05–10	°C	16	38.09 ± 0.52	10	38.43 ± 0.31	<0.05
RT 10–15	°C	16	38.07 ± 0.31	10	38.38 ± 0.48	>0.05
RT 15–20	°C	16	38.15 ± 0.39	10	38.35 ± 0.34	>0.05

**Table 3:** Differences (*α* = 0.05) in drug doses, clinical and physiological parameters between male and female snow leopards (*Panthera uncia*).

Variables	Unit	Female		Male		*p*-value
		*n*	Mean ± SD	*n*	Mean ± SD	
Drug doses						
Xylazine HCL	mgkg^−1^	15	2.041 ± 0.39	11	1.796 ± 0.41	>0.05
Ketamine HCL	mgkg^−1^	15	6.548 ± 0.98	11	6.519 ± 0.89	>0.05
Yohimbine	mgkg^−1^	15	0.147 ± 0.034	11	0.146 ± 0.02	>0.05
Clinical parameters						
Induction time	Minute	15	3.53 ± 1.96	11	4.27 ± 1.55	>0.05
Recumbency period	Minute	15	69.73 ± 13.89	11	72.00 ± 20.32	>0.05
Recovery following reversal	Minute	15	25.00 ± 7.91	11	24.82 ± 6.12	>0.05
Physiological parameters						
RR 05 (on approach)	/minute	15	31.67 ± 4.53	11	29.27 ± 3.97	>0.05
RR 05–10	/minute	15	29.93 ± 3.21	11	29.36 ± 3.35	>0.05
RR 10–15	/minute	15	27.60 ± 2.72	11	29.64 ± 3.02	>0.05
RR 15–20	/minute	15	28.40 ± 2.06	11	30.45 ± 2.91	>0.05
RR 20–25	/minute	15	29.73 ± 2.52	11	31.00 ± 2.86	>0.05
RR 25–30	/minute	15	29.53 ± 1.64	11	30.09 ± 2.21	>0.05
HR 05 (on approach)	/minute	15	74.93 ± 13.35	11	72.09 ± 10.12	>0.05
HR 05–10	/minute	15	74.20 ± 12.52	11	71.36 ± 7.87	>0.05
HR 10–15	/minute	15	73.67 ± 11.96	11	71.73 ± 7.74	>0.05
HR 15–20	/minute	15	75.33 ± 13.09	11	70.27 ± 6.45	>0.05
HR 20–25	/minute	15	73.80 ± 11.16	11	70.09 ± 6.31	>0.05
RT (on approach)	°C	15	38.27 ± 0.38	11	38.05 ± 0.65	>0.05
RT 05–10	°C	15	38.25 ± 0.47	11	38.19 ± 0.49	>0.05
RT 10–15	°C	15	38.27 ± 0.42	11	38.08 ± 0.36	>0.05
RT 15–20	°C	15	38.25 ± 0.35	11	38.19 ± 0.42	>0.05

## Discussion

We found that a combination of ketamine-hydrochloride (administered at 6.535 ± 0.93 mgkg^−1^ body weight) and xylazine-hydrochloride (administered at 1.937 ± 0.41 mgkg^−1^ body weight) were adequate mixture/combinations to achieve the safe immobilization of free-ranging snow leopards for managing emergency such as rescue operations and health assessment. These two drug combinations have previously been used on snow leopards (15, 19, 21–25). In the current study, ketamine dose rate was found to be lower as compared to previous research findings (15, 18, 21, 22, 25) and almost similar to other studies (19, 24). Similarly, for xylazine dose obtained in the study was found to be lower than the study done previously (15) and similar to other previous studies (19, 25) and higher than studies conducted before (21, 22, 24, 26). We found that ketamine-xylazine drug combinations at an average ratio of 3.5:1 (3.5 mg ketamine is required per 1 mg of xylazine) was effective in inducing smooth and rapid anesthesia for free-ranging snow leopards for emergency management. We did note that a significantly higher ketamine dose was required for young animals, as an additional drug was required than the default dose for capturing the young animals, but there were no differences in effective dose between the sexes.

For the clinical parameters, it was found that on average induction time was complete within ten minutes which was similar to the previous findings (15, 24). No significant differences were found for clinical parameters between adult and young snow leopards, and the same was observed in the case of males and females.

All recorded physiological parameters (heart rate, respiratory rate, and rectal temperature) were all within the normal limits (15). There is no significant difference found across sex for physiological parameters.

Yohimbine to reverse xylazine was effective at an average dose rate of 0.147 ± 0.03 mgkg^−1^ and was similar to previous findings (25). Yohimbine has long been used as an antagonist for xylazine-induced sedation and in other wild cats to hasten recovery (26). Though there were no significant differences found for the dose rate of yohimbine across age and classes.

In summary, this study presents a comprehensive clinical and physiological assessment of free-ranging snow leopards (*Panthera uncia*) immobilized with a ketamine-xylazine drug combination. The findings contribute valuable insights to snow leopard conservation efforts by providing consistent data from a significant number of animals immobilized with various drug mixtures in their natural habitat.

The physiological variables and drug doses documented in this study for the ketamine-xylazine mixture offer crucial information for managing field emergencies such as rescue operations and health assessments. These findings serve as reference values for physiological parameters that may be helpful in field emergency rescues. However, the doses should be modified according to animal conditions and situations.

Furthermore, the comprehensive evaluation of physiological responses provides a foundation for future research and clinical practice in snow leopard conservation. By establishing primary values for vital signs and drug dosages, this study empowers conservationists and veterinarians to make informed decisions when managing the health of snow leopards in their natural habitat. However, more work is suggested in this direction.

## Data Availability

The original contributions presented in the study are included in the article/Supplementary material, further inquiries can be directed to the corresponding author.
